# Development and Validation of a Clinical Prediction Model for Venous Thromboembolism Following Neurosurgery: A 6-Year, Multicenter, Retrospective and Prospective Diagnostic Cohort Study

**DOI:** 10.3390/cancers15225483

**Published:** 2023-11-20

**Authors:** Deshan Liu, Dixiang Song, Weihai Ning, Yuduo Guo, Ting Lei, Yanming Qu, Mingshan Zhang, Chunyu Gu, Haoran Wang, Junpeng Ji, Yongfei Wang, Yao Zhao, Nidan Qiao, Hongwei Zhang

**Affiliations:** 1 Department of Neurosurgery, Sanbo Brain Hospital, Capital Medical University, Beijing 100093, China; alfredliu@mail.ccmu.edu.cn (D.L.); songdixiang@mail.ccmu.edu.cn (D.S.); sanboocean@163.com (W.N.); guoyuduo@hotmail.com (Y.G.); leiting@mail.ccmu.edu.cn (T.L.); quyanming@sina.com (Y.Q.); zhangms7606@sina.com (M.Z.); guchunyu0705@sina.com (C.G.); whr0517@126.com (H.W.); jpji1991@163.com (J.J.); 2 Department of Neurosurgery, Huashan Hospital, Shanghai Medical School, Fudan University, Shanghai 200030, China; eamns@hotmail.com (Y.W.); zhaoyaohs@vip.sina.com (Y.Z.)

**Keywords:** venous thromboembolism, neurosurgery, risk assessment, risk factors, primary prevention

## Abstract

**Simple Summary:**

The neurosurgery patient population belongs to the moderate- to high-risk venous thromboembolism (VTE) population. There is no specific clinical prediction model for the incidence of postoperative VTE in neurosurgery. This study developed a comprehensive model by combining specific laboratory biomarkers, a large sample size, and various perioperative variables to standardize primary VTE prevention, and the model exhibited strong predictive performance across multiple validation cohorts. Neurosurgeons can utilize this model to make informed decisions regarding appropriate VTE primary prevention strategies during the early postoperative period.

**Abstract:**

Background: Based on the literature and data on its clinical trials, the incidence of venous thromboembolism (VTE) in patients undergoing neurosurgery has been 3.0%~26%. We used advanced machine learning techniques and statistical methods to provide a clinical prediction model for VTE after neurosurgery. Methods: All patients (n = 5867) who underwent neurosurgery from the development and retrospective internal validation cohorts were obtained from May 2017 to April 2022 at the Department of Neurosurgery at the Sanbo Brain Hospital. The clinical and biomarker variables were divided into pre-, intra-, and postoperative. A univariate logistic regression (LR) was applied to explore the 67 candidate predictors with VTE. We used a multivariable logistic regression (MLR) to select all significant MLR variables of MLR to build the clinical risk prediction model. We used a random forest to calculate the importance of significant variables of MLR. In addition, we conducted prospective internal (n = 490) and external validation (n = 2301) for the model. Results: Eight variables were selected for inclusion in the final clinical prediction model: D-dimer before surgery, activated partial thromboplastin time before neurosurgery, age, craniopharyngioma, duration of operation, disturbance of consciousness on the second day after surgery and high dose of mannitol, and highest D-dimer within 72 h after surgery. The area under the curve (AUC) values for the development, retrospective internal validation, and prospective internal validation cohorts were 0.78, 0.77, and 0.79, respectively. The external validation set had the highest AUC value of 0.85. Conclusions: This validated clinical prediction model, including eight clinical factors and biomarkers, predicted the risk of VTE following neurosurgery. Looking forward to further research exploring the standardization of clinical decision-making for primary VTE prevention based on this model.

## 1. Introduction

Venous thromboembolism (VTE) after different elective operations occurred at a rate of 0.8% based on the issued literature [1]. Moreover, the incidence of VTE in patients undergoing neurosurgery without prophylaxis related to VTE was 2.3%~43% [2,3,4,5,6]. VTE incidence after a neurosurgical procedure is very high, and deep venous thrombosis (DVT) causes bad consequences, such as increasing hospital costs and overall morbidity and mortality of patients [7]. Pulmonary embolism (PE), as part of VTE, carries a high mortality [8]. The previous study on postoperative VTE in neurosurgery shows risk factors that include surgery for malignancy, duration of surgery, increased age, lower limb paralysis, infection, surgery time ≥ 4 h, and septicemia [1,6,9,10,11,12]. However, few neurosurgical studies have combined specific laboratory biomarkers, large sample size, and numerous perioperative variables. The incidence of postoperative VTE in neurosurgical patients should be thoroughly evaluated according to the pre-, intra-, and postoperative risk factors. Only by quantifying the incidence of postoperative VTE can we provide a more precise primary prevention of VTE in neurosurgery patients.

VTE risk assessment models are commonly used to quantify the risk of VTE after nonorthopedic surgery. However, there is no universally accepted model, and many physicians use a comprehensive assessment. Some models are widely used, for example, the modified Caprini risk assessment model (i.e., ACCP modified Caprini score) [13]. Although this model has been verified, it only applies to patients undergoing general, abdominal, and pelvic surgery [13,14,15]. Moreover, the extensive entries in the Caprini risk assessment model reduce its practicality. For clinicians, employing this model might be time-consuming. This assessment dealt only with stroke relating to neurosurgery and did not include laboratory tests such as D-dimer [16]. The Wells clinical model for predicting pretest probability for deep-vein thrombosis is widely used and was simplified by stratifying patients into either low- or high-risk populations [17]. The weight of clinical symptoms was high, but most patients with DVT have no clinical symptoms. The Padua risk assessment model is a scale developed by Padua University based on a retrospective review of internal medicine patients. It is primarily intended for internal medicine patients [18]. The Khorana scale is mainly used to evaluate outpatients undergoing chemotherapy and is not suitable for surgical inpatients [19]. The four-element risk assessment model was designed by Woller in 2011 for inpatients in internal medicine [20]. There is no specific clinical prediction model for the incidence of postoperative VTE in neurosurgery. The neurosurgery patient population belongs to the moderate- to high-risk VTE population [13,21]. Relevant research has not fully elucidated how to refine the moderate to high-risk VTE population of neurosurgery. Present scales do not yet capture the specific characteristics of neurosurgery patients.

Therefore, we initiated a multicenter study that included neurosurgical populations from four cohorts. The study aimed to explore the preoperative, intraoperative, and postoperative factors of postoperative VTE in neurosurgery to construct a clinical prediction model with the aim of standardizing the primary prevention of VTE after neurosurgery.

## 2. Materials and Methods

This report applies transparent reporting of a multivariate prediction model for individual prognosis or diagnosis (TRIPOD) guidelines (Supplementary Materials S2) [22].

### 2.1. Study Design and Participants

Data collection was primarily obtained from a retrospective hospital study to develop and validate a clinical risk assessment model to predict DVT. This development and retrospective internal validation cohorts were formed by computer databases and electronic medical records for 5867 patients. Included were all patients who underwent neurosurgery between May 2017 and April 2022 at the Department of Neurosurgery at the Sanbo Brain Hospital of Capital Medical University. The collected data were randomly divided into two cohorts. This development cohort (n = 4401) was to develop this clinical model, and the other retrospective internal validation cohort (n = 1466) was to validate it. Inclusion criteria were (1) age > 18 years, (2) underwent neurosurgical procedures during hospitalization, and (3) underwent preoperative ultrasound examination. Exclusion criteria were (1) a patient with obvious bacterial or viral infection within the past two weeks before admission, (2) a patient with VTE within the past three months before surgery, (3) a patient with anticoagulant therapy (direct oral anticoagulants, low molecular weight heparin) that was administered continuously or intermittently before admission, and (4) a patient with a prior coagulation dysfunction. Then, our neurosurgical team collaborated closely with the Hospital Network Information Department to integrate the developed VTE clinical prediction model into the hospital’s order system. The prospective validation of the VTE clinical prediction model commenced on 7 March 2023 and concluded on 25 May 2023. This cohort served as an internally prospective validation cohort, adhering to the same inclusion and exclusion criteria mentioned previously. Informed consent was obtained during preoperative conversations in the hospital. Simultaneously, we conducted an external validation of our neurosurgical VTE clinical prediction model in partnership with the Department of Neurosurgery at Huashan Hospital. Huashan Hospital initiated a clinical study [23] on VTE in December 2019, with prospective data collection for the variables included in our VTE model and comprehensive recording of outcome events. The same inclusion and exclusion criteria mentioned earlier were applied to this cohort. Data from all patients were utilized according to the principles of the Helsinki Declaration and summed for this clinical model. This study was approved by the ethics committee of our institution (SBNK-YJ-2021-022-01). The prospective validation of the model has been registered on clinicaltrials.gov (NCT05860790).

### 2.2. Defining VTE and Sample Size Considerations

Real-time B-mode compression ultrasonography was performed on all patients in the cohorts to ensure that there was no patient with preoperative DVT. If thrombosis was detected, the patient needed to be ruled out because our objective was to predict the incidence of postoperative VTE. Therefore, it was important to ensure that there was no preexisting thrombosis before surgery to have predictive significance in detecting the incidence of postoperative VTE. Senior doctors from these two medical centers determined postoperative ultrasonography according to the symptoms, physical examination, or coagulation parameters of patients. The patients were diagnosed with DVT according to the guidelines [24]. When hospitalized patients presented with clinical manifestations suggestive of PE and exhibited hemodynamic instability, particularly in the presence of symptoms of DVT or confirmed DVT, physicians from both medical centers would immediately proceed with performing a computed tomography pulmonary angiography (CTPA) examination to confirm the diagnosis of PE. The primary outcome of this study was objectively confirmed VTE after neurosurgery during hospitalization. It combined proximal or distal DVT of the leg, upper limb DVT, or PE. Patients did not routinely receive anticoagulant prophylaxis after neurosurgery at these two medical centers. When ultrasonography detected a proximal DVT, the patient was required to be subcutaneously injected with low-molecular-weight heparin (LMWH).

The aim of our study was to develop and validate a predictive model for postoperative VTE in neurosurgery. In previous studies, if the events per variable (EPV) are too low, the frequency distribution of the regression coefficients for the measured variables may be more dispersed. The distribution of regression coefficients deviates from normal distribution, and the relative percentage bias also increases. To ensure the validity of the model, we chose a minimum event per variable of 10 as the criterion to determine the sample size for each variable. The 10 EPV is a widely applied principle, suggesting that the number of positive events should be at least 10 times the number of predictor variables, currently a classical rule for sample sizes in clinical prediction models [25,26,27]. We included 21 variables in the multivariable logistic regression (MLR) analysis. Therefore, a minimum sample size of 210 VTE events is needed for the development phase. For external validation of the predictive model, a minimum sample size of 100 VTE events is recommended [28]. We combined data from literature reviews on incidence rates (2.3–43%) and selected a lower postneurosurgical VTE incidence rate (5%) to maximize our final planned sample size, ensuring the accuracy and maximum precision of the model’s regression coefficients. This was aimed at reducing overfitting or underfitting of the model. Consequently, the minimum sample sizes planned for the development and external validation sets were 4200 and 2000, respectively. Actual sample sizes for the development and validation sets were 4401 and 2301, respectively.

### 2.3. Collection of Variables

In order to comprehensively evaluate the occurrence of postoperative VTE, we conducted a literature review and organized discussions among professionals in neurosurgery, anesthesiology, pathology, ultrasound departments, and other relevant fields. Combining the data available from preoperative, intraoperative, and postoperative electronic medical records in our hospital, we selected 67 candidate predictive factors and formulated a case report form (CRF). The preoperative variables included clinical indices and laboratory results. The clinical indices consisted of the age; sex of the patients; Karnofsky performance status scale (KPS); body mass index (BMI); preoperative hospital stay; American Society of Anesthesiology (ASA) score; medical history based on the ICD-10 code (including hypertension, diabetes, hyperlipidemia, nephropathy (a combination of chronic nephritic syndrome (N03)), hepatopathy (a combination of liver fibrosis and cirrhosis (K74), chronic viral hepatitis (B18) and toxic liver disease(K71)), and varicosity(I83); patient in wheelchairs or bedridden and patient with intracranial aneurysm; intracranial arteriovenous malformations (AVM); carotid artery stenosis; trauma; hydrocephalus; spinal vascular malformation; epilepsy; trigeminal neuralgia; hemifacial spasm; and brain abscess. The blood laboratory results before neurosurgery contained D-dimer, activated partial thromboplastin time (APTT), fibrinogen, prothrombin activity, thrombin time, prothrombin time, platelets, white blood cells, hemoglobin, Na, Cl, K, total cholesterol, triglyceride, low-density lipoprotein (LDL), alanine aminotransferase (ALT), uric acid, and serum homocysteine. The details of the reagents used in the laboratory can be found in Table S1 in Supplementary Materials S1. The intraoperative variables included the operation duration, bleeding volume, operation position (prone position), operation level, anesthesia mode, and operation location (cerebellar hemisphere, lateral ventricle, third ventricle, fourth ventricle, cavernous sinus tumor, cranial base, intraspinal, and intramedullary). The postoperative variables comprised the highest D-dimer within 72 h after surgery, disturbance of consciousness on the second day after surgery, and high dose of mannitol (the amount of 20% mannitol administered ≥ 250 mL intravenous drip q.8h. for three days), central venous catheterization (CVC), lumbar cisterna drainage, hemiplegia and paraplegia, and histopathological type of tumor according to the World Health Organization classification (malignant and secondary tumor, pituitary tumor, craniopharyngioma, germinoma, and acoustic neuromas) [29,30]. In the prospective internal validation cohort and the external validation cohort, we collected baseline characteristics of the patients, along with the variables that were ultimately included in the clinical prediction model for VTE.

### 2.4. Statistical Analysis

Principal component analysis (PCA) was used to describe the overall data distribution [31]. Due to the inclusion of numerous variables, we utilized the PCA method to visualize the data, enabling the identification of patterns and structures within the data and facilitating exploration of the relationships among variables. The contribution, outlier, and major source of variance, together with visualization of the reduction in dimensionality for each datum, can be provided by this method in the entire retrospective study cohort. MissForest were used for missing value imputation. Additionally, the box plot showed the D-dimer difference in patients with VTE (total VTE, distal DVT, proximal DVT, upper limb DVT, and PE) and without VTE at different times during the neurosurgical perioperative period by Wilcox test or one-way analysis of variance (ANOVA). Calculated *p*-values < 0.05 were considered statistically significant differences. To randomize the allocation of samples into the development and retrospective internal validation sets, we employed a simple random sampling approach without replacement. Subsequently, by randomly shuffling this sequence, we disrupted the original order of the samples. Based on the predefined ratios of the development set and retrospective internal validation set, we calculated the number of samples for the development set. Finally, using the shuffled indices, we partitioned the dataset into temporary development and retrospective internal validation sets. The temporary development set consisted of the specified number of samples, while the temporary validation set contained the remaining samples. For model development and validation, the occurrence of VTE after neurosurgery during hospitalization was considered a binary outcome. The variable selection process for model inclusion consisted of four steps. In the first step, a univariate logistic regression (LR) was applied to explore the 67 candidate predictors with VTE in the development cohort. In the second step, based on the magnitude of the Wald statistic of LR, variables that were statistically significant in the univariate LR analysis and applicable to real clinical scenarios were selected for inclusion in the MLR analysis. Due to the large sample size, we were able to include all 21 candidate predictive factors in a multivariable logistic regression analysis to effectively control potential confounding factors. Ten-fold cross-validation evaluated the robustness of the MLR model. In the third step, the statistically significant variables from the MLR analysis were subjected to random forest (RF) analysis to rank their importance [32]. During training, decision trees were used to split the nodes based on the importance of the features. Consequently, an RF quantifies the significance of each feature by the number of times a split occurs on nodes across all decision trees. These statistical details, when aggregated across all trees, yield the relative importance of each feature. Accuracy selected the optimal RF model using the largest value (The final value used for the RF model was 7). In the fourth step, within the range of variables determined by the RF analysis, suitable variables were selected for inclusion in the final clinical prediction model based on their importance and clinical applicability. MLR and LR models calculated the odds ratio with the corresponding 95% CI and *p*-values. 

The receiving operating characteristics (ROC) curve was used to assess the discrimination of the MLR model. We used the maximum Youden index to select the cutoff [33]; the higher the Youden index, the greater the credibility. The maximum value represents the optimal diagnostic threshold. Then, we used a calibration plot to determine the calibration between the actual and predicted outcome probabilities. Decision-curve analysis (DCA) was applied to assess the clinical benefit. R packages (randomForest, FactoMineR, rms) in R software (Version 4.2.0) were used to form the PCA, RF, and MLR models [34,35].

## 3. Results

### 3.1. Entire Retrospective Study Cohort

**Figure 1:**
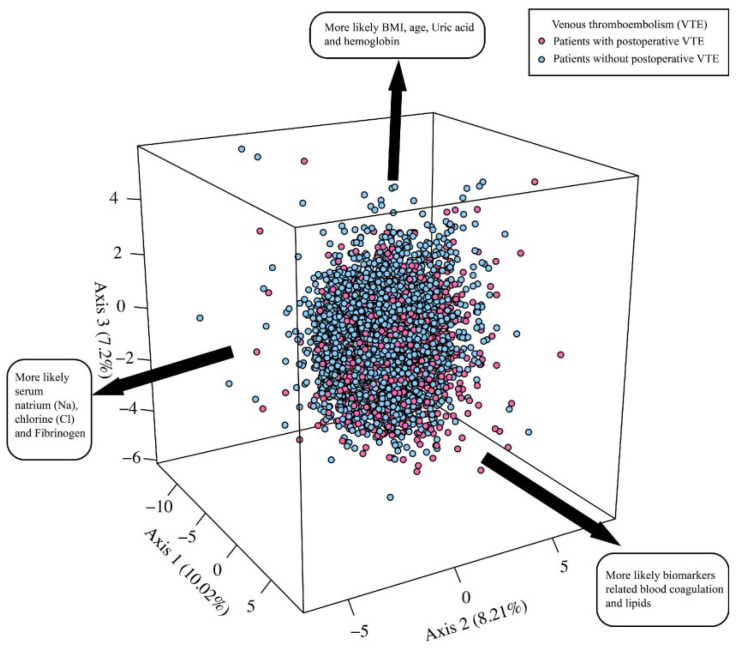
The global profiles of the principal component analysis (PCA) based on the distribution of 67 variables along the three most important axes. Patients with venous thromboembolism (VTE) (hot pink circles) and without VTE (light blue circles) are represented on this graph. Each axis denotes a principal component from the PCA method. The percentage of variance of each axis is given within parenthesis. BMI, body mass index.

We retrieved the medical records of 7795 patients who had complete ultrasound examinations between May 2017 and April 2022 at the Department of Neurosurgery of the Sanbo Brain Hospital of Capital Medical University. A total of 6048 patients were initially included after removing 1312 patients without surgery and 435 patients whose ages did not meet the inclusion criteria. Consequently, we excluded 181 from included medical records according to the exclusion criteria (Figure S1a in Supplementary Materials S1). The PCA analysis comprehensively analyzed the full picture of 5867 patients assessed for eligibility based on the distribution of 67 variables along the three most essential axes (Figure 1). Figure 1 also integrates the patients with VTE (hot pink circles) and the patients without VTE (light blue circles). Using this method, the overall ability of the three axes to explain differences between VTE and the absence of VTE reached 25% cumulated. The first axis (10.40% of explained variance) typically shows biomarkers related to blood coagulation and lipids. The second axis (8.54% of the explained variance) shows serum Na, Cl, and fibrinogen. The third axis (7.32% of the explained variance) shows BMI, age, uric acid, and hemoglobin. Figure S2 in Supplementary Materials S1 presents the importance of the contribution of variables.

Randomization proportion for the development and retrospective internal validation sets was 3:1. Table 1 illustrates the randomization results and provides information on the baseline demographic characteristics and primary outcome (VTE events) for the study populations in each cohort, including the variables included.

**Figure 2:**
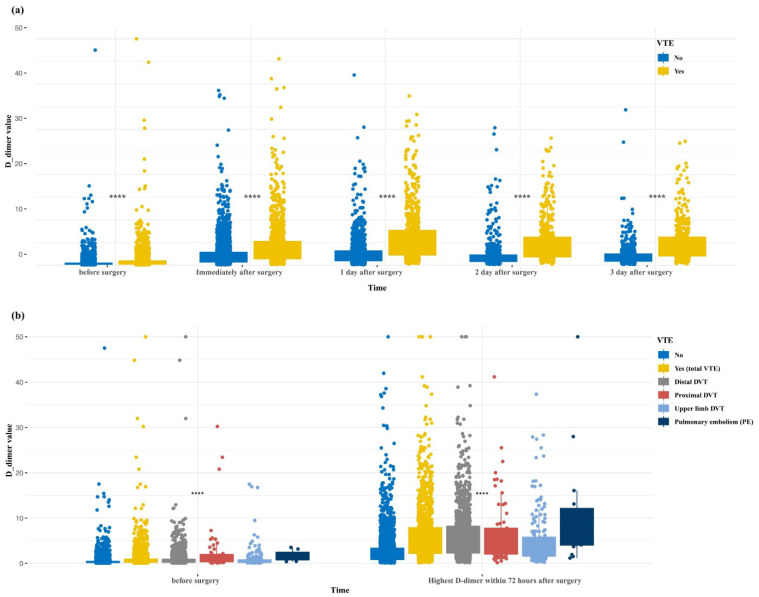
(**a**) D-dimer values (µg/mL) before and after neurosurgical surgery. **** indicates *p* < 0.0001, wilcox.test. (**b**) D-dimer values (µg/mL) of the patients without VTE, with VTE, distal deep venous thrombosis (DVT), proximal DVT, upper limb DVT, and PE before and after neurosurgical surgery. **** indicates *p* < 0.0001, one-way analysis of variance (ANOVA). VTE, venous thromboembolism. DVT, deep-vein thrombosis.

Table S2 provides a comprehensive description of the surgical indications for all patients in each neurosurgery cohort. In the entire retrospective cohort, univariable logistic regression was utilized to calculate the odds ratios (ORs) representing the correlation between various types of neurosurgical procedures and the occurrence of postoperative VTE; it was found that cerebrovascular neurosurgery (OR = 1.596, 95% CI [1.261, 2.019]) for aneurysms, arteriovenous malformations, stroke, and other related conditions is a risk factor for postoperative VTE (Table S3 in Supplementary Materials S1). Figure S1b,c in Supplementary Materials S1 display the flowcharts of the prospective validation cohort and external validation cohort, respectively. Additionally, D-dimer in a patient with VTE is statistically significantly different from patients without VTE no matter on preoperative, immediately after surgery, on the first, second or third postoperative day (Figure 2a). Among patients without VTE, with VTE, distal DVT, proximal DVT, upper limb DVT and PE, the median D-dimer value of patients diagnosed with postoperative PE was the highest regardless of the preoperative or early postoperative period (Figure 2b).

### 3.2. Model Development

Table 2 displays the result of the calculation of the ORs of 67 candidate predictors related to VTE for the univariate logistic regression of the development cohort in the first step. The risk factors with *p*-values less than 0.01 are described in the following text. Regarding preoperative clinical risk factors, demographic variables such as age (OR = 1.047, 95% CI [1.041, 1.052]) are important risk factors. Patient admission statuses like KPS (OR = 0.985, 95% CI [0.982, 0.988]) and ASA (OR = 2.706, 95% CI [2.238, 3.271]) are also significant postoperative risk factors. Certain preoperative diagnoses such as hypertension (OR= 1.699, 95% CI [1.446, 1.996]) and intracranial aneurysm (OR = 1.980, 95% CI [1.442, 2.718]) also influence the occurrence of VTE after neurosurgical procedures. Preoperative laboratory variables proven to be a risk factor included D-dimer (OR = 1.428, 95% CI [1.332, 1.531]), APTT (OR = 0.919, 95% CI [0.898, 0.940]), and fibrinogen (OR = 1.2, 95% CI [1.116, 1.29]). Interestingly, we found that as the uric acid levels (OR = 0.998, 95% CI [0.997, 0.998]) increased, the incidence rate of VTE decreased.

Duration of the operation (OR = 1.003, 95% CI [1.002, 1.003]) and bleeding volume (OR = 1.000, 95% CI [1.000, 1.001]) are risk factors for intraoperative variables in VTE after neurosurgery.

Postoperatively, the highest D-dimer within 72 h after surgery (OR = 1.208, 95% CI [1.184, 1.233]) and the disturbance of consciousness (OR = 3.363, 95% CI [2.617, 4.321]) are significant risk factors. Some tumor pathologies such as malignant tumors (OR= 1.287, 95% CI [1.119, 1.480]) and craniopharyngioma (OR = 1.948, 95% CI [1.499, 2.531]) also pose risks for VTE. However, the diagnosis of pituitary tumors (OR = 0.467, 95% CI [0.353, 0.617]) decrease the incidence of VTE. Additionally, some postoperative medical orders like high dose of mannitol (OR = 1.447, 95% CI [1.253, 1.670]), CVC (OR = 1.346, 95% CI [1.166, 1.554]), and lumbar cisterna drainage (OR = 1.472, 95% CI [1.2, 1.806]) hold statistical significance. In the second step, significant variables identified through univariate LR were incorporated into the MLR model based on their practical clinical availability in real-world scenarios.

**Figure 3:**
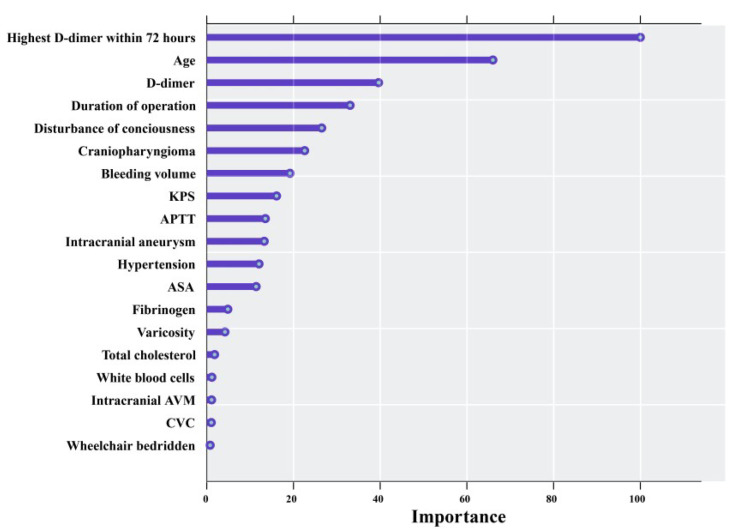
Random forest model calculation showing the importance of the risk factors that were statistically significant in the multivariable logistic regression (MLR) analysis. This model was used for the selection of multiple variables, ranking them based on the contribution of each variable in the random forest decision trees used for node splitting. The figure ranks the importance of each risk factor of postoperative risk VTE in the random forest model in order from greatest to smallest. KPS, Karnofsky performance status scale. ASA, American Society of Anesthesiology score. APTT, activated partial thromboplastin time. CVC, central venous catheterization. Highest D-dimer within 72 h is the highest value selected from the data tested immediately after surgery, the first day after surgery, the second day after surgery, and the third day after surgery. The third variable counted from the top down was the value of preoperative D-dimer.

Table 2 also illustrates the results of the MLR model. We found that age, duration of operation, D-dimer, high dose of mannitol, APTT, craniopharyngioma, and disturbance of consciousness were statistically significant variables in the MLR model. In the third step, we included the statistically significant risk factors identified through MLR analysis into the RF analysis. Figure 3 presents the importance ranking of statistically significant risk factors identified in the MLR analysis.

**Figure 4:**
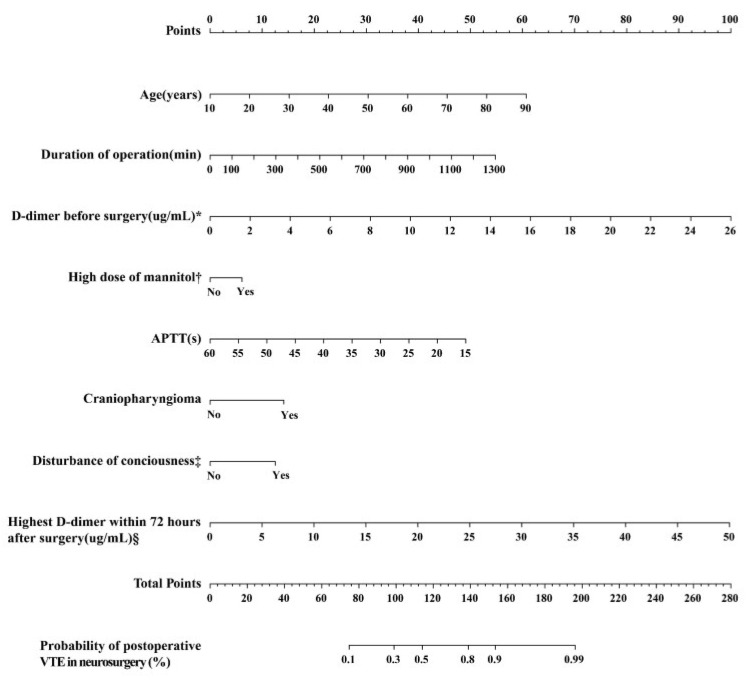
Nomogram to predict postoperative VTE in neurosurgery. VTE, venous thromboembolism. APTT, activated partial thromboplastin time. * This indicates the value of preoperative D-dimer. † High dose of mannitol represents the amount of 20% mannitol administered ≥ 250 mL intravenous drip q.8h. for three days after surgery. ‡ Disturbance of consciousness on the second day after surgery. § Highest D-dimer within 72 h is the highest value selected from the data tested immediately after surgery, the first day after surgery, the second day after surgery, and the third day after surgery.

The three most important factors identified in the RF analysis are highest D-dimer within 72 h after surgery, age, and D-dimer before surgery. In the fourth step, based on the results of the RF analysis and the real-world clinical scenario, we selected eight variables to be included in the final model and constructed a nomogram (Figure 4).

### 3.3. Model Validation

**Figure 5:**
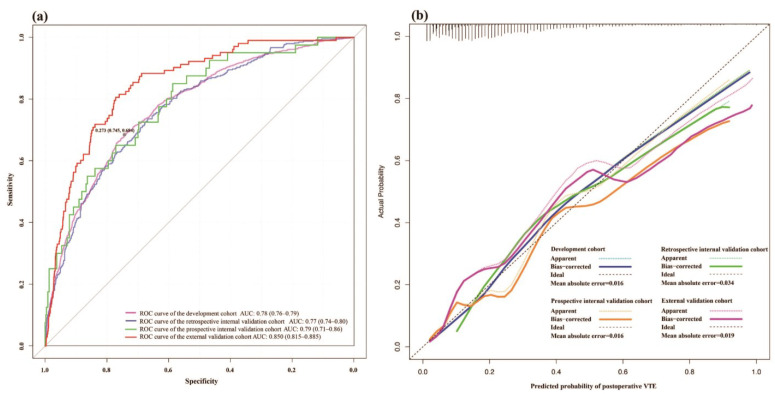
(**a**) The receiving operating characteristics (ROC) curves of the nomograms. ROC curves of the nomograms in the development, retrospective internal validation, prospective internal validation, and external validation cohorts. The area under the curve (AUC) values were calculated for the training, retrospective internal validation, and prospective internal validation cohorts as follows: 0.78 (95% CI: 0.76–0.79), 0.77 (95% CI: 0.74–0.80), and 0.79 (95% CI: 0.71–0.86), respectively. The external validation set exhibited the highest AUC value (0.85 (95% CI: 0.815–0.885)). The gray dot represents the best cutoff value. (**b**) Calibration curves of the nomogram prediction in the development, retrospective internal validation, prospective internal validation, and external validation cohorts. The actual and predicted probability of postoperative VTE is plotted on the Y-axis and X-axis. AUC, area under the receiver operating characteristic curve. ROC, receiver operating characteristic. VTE, venous thromboembolism.

The ROC curves indicate similar discrimination of the MLR model in the development, retrospective internal, and prospective internal validation cohorts. Additionally, the model exhibited even higher discriminative ability in the external validation cohort. The AUC (area under the curve) values were calculated for the training, retrospective internal validation, and prospective internal validation cohorts as follows: 0.78 (95% CI: 0.76–0.79), 0.77 (95% CI: 0.74–0.80), and 0.79 (95% CI: 0.71–0.86), respectively. The external validation set exhibited the highest AUC value (0.85 (95% CI: 0.815–0.885)) (Figure 5a). The ten cross-validated AUC for this model in the development cohort was 0.77 (95% CI: 0.77–0.78). Figure 5b illustrates calibration plots overlapped with the ideal line in all the datasets included.

**Figure 6:**
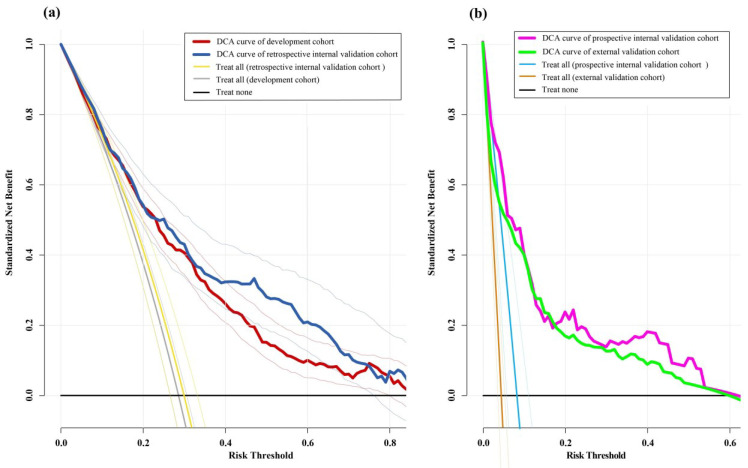
(**a**) Decision-curve analysis for postoperative VTE in the development and retrospective internal validation cohort. Standardized net benefit and 95%CI, shown in red (development) and blue (retrospective internal validation) lines, was calculated as the true-positive rate minus the weighted false-positive rate for the clinical benefit of the model across the full range of risk threshold. (**b**) Decision-curve analysis for postoperative VTE in prospective internal validation and external validation cohort. Standardized net benefit, shown in pink (prospective internal validation) and green (external validation) lines, was calculated as the true-positive rate minus the weighted false-positive rate for the clinical benefit of the model across the full range of risk threshold. The risk threshold denotes the prediction value for the risk of postoperative VTE. The line of treating all denotes that all patients are both given the thromboprophylaxis. The line of treating none denotes that no patient is given the thromboprophylaxis. DCA, decision-curve analysis.

These results indicate adequate agreement of this nomogram with the actual observations. Decision-curve analysis for each cohort showed that the model had greater clinical utility for indications for thromboprophylaxis than the treatment strategies for all or none (Figure 6a,b).

Consequently, an online calculator based on the final model was generated by integrating the weight of each factor associated with postoperative VTE. The VTE calculator website is available here (https://proofvte.shinyapps.io/sanboneurosurgery/, (accessed on 5 December 2022)).

## 4. Discussion

In this study, a nomogram was developed and validated to form an online calculator to predict VTE after neurosurgery. We used this new method to predict the risk of VTE after neurosurgery, which is beneficial in screening out the relatively low-risk population and avoiding bleeding caused by excessive use of chemoprophylaxis to prevent VTE. According to this nomogram, drug application also reduces the risk of postoperative VTE and potentially fatal PE or proximal thrombosis in the high-risk population of VTE.

Our open nomogram is based on the inclusion of patients with several neurosurgical indications (refer to Table S2 in Supplementary Materials S1). Furthermore, the predictive ability of the model performed well in both internal and external validation. Thus, it is more suitable for evaluating VTE after neurosurgery from the perspective of applicability.

When reviewing the literature for our clinical practice and study, advancing age is a strong prognostic clinical driver. Moreover, the weight in the duration of neurosurgical operation on the occurrence of postoperative VTE is also very large, which can be demonstrated from prior studies [11,36]. Neurosurgical operation is generally longer than surgery in other fields, increasing the incidence of VTE after neurosurgery. Intraoperative physical anticoagulation should be studied to minimize the risk of VTE due to the prolonged duration of operation [37]. For elderly patients and those with prolonged surgical durations, neurosurgeons should be particularly vigilant in monitoring postoperative occurrences of VTE. Compared to neurosurgery for brain tumors, functional neurosurgery, and spinal neurosurgery, cerebrovascular neurosurgery carries a higher postoperative risk of VTE. In our study, it is a confirmed risk factor for postoperative VTE occurrence. This may be attributed to several factors within the field of cerebrovascular neurosurgery. Patients in this subspecialty often present with more severe medical conditions, necessitating extended periods of bed rest. Additionally, their vascular health is generally compromised, especially concerning arterial vessels. Patients undergoing cerebrovascular neurosurgery frequently exhibit preexisting risk factors for VTE, such as smoking, hypertension, diabetes, hypercholesterolemia, and hyperlipidemia [38]. Consequently, these factors collectively contribute to the heightened risk of VTE in this patient population. This should draw the attention of neurosurgeons specializing in cerebrovascular procedures.

The highest D-dimer value within 72 h after surgery is the most critical laboratory biomarker among all variables we included. It has been proven in the corresponding clinical literature that it can predict VTE [39]. Through our large sample study, the pre- and postoperative D-dimer values were statistically significant risk factors for postoperative VTE. It would be valuable to test the value of D-dimer before surgery. An elevated D-dimer in plasma indicates simultaneous activation of coagulation and fibrinolysis. The most important part is the balance between coagulation and fibrinolysis. Hyperactivity on one side may lead to an elevated D-dimer value. However, hyperactivity on both sides of the scale may lead to normal D-dimer values. Hence, it is not blindly assumed that normal D-dimer levels can exclude the occurrence of postoperative VTE. Second, an elevated D-dimer should be analyzed according to the specific clinical situation. Due to its low positive predictive value, a high D-dimer level should be taken into account for some other diseases or physiological conditions, such as severe inflammatory diseases, cancer, and pregnancy [40,41,42]. Relying solely on postoperative D-dimer levels to determine the need for lower limb ultrasonography is possibly incomplete, as we incorporate multiple factors to comprehensively predict VTE. To ensure that D-dimer levels could predict postoperative VTE as much as possible, D-dimer values were introduced at two time points. The age variable was introduced to correct the error caused by advanced age.

In neurosurgery, the frequency of the postoperative dehydrating drug mannitol is very high. It is often used to treat brain edema and intracranial hypertension and prevent the occurrence of cerebral hernia. As a tissue dehydration drug, mannitol has adverse effects, including electrolyte disturbances and excessive inappropriate diuresis leading to blood stasis, which is critical in the Virchow triad [43,44]. Our study demonstrated that the application of mannitol in a high dose (daily dose greater than 0.15 kg/24 h) after surgery aggravates the occurrence of VTE. There is a paucity of studies exploring the association between high doses of mannitol and VTE. Only a few studies suggest that it may cause VTE due to agglutination and irreversible creation of red blood cells as a source of embolism [45,46]. Neurosurgeons, while considering dehydration for intracranial pressure reduction, should also be cautious as high doses of mannitol may increase the risk of VTE.

Similarly, APTT is a sensitive screening test of the endogenous coagulation system, which mainly reflects the level of endogenous coagulation. In our study, the shortening of APTT increased the probability of VTE and had a large weight compared to other factors that were included. It was found that when the procoagulant enters the blood and the activity of the coagulation factors increases, the APTT is reduced [47]. The D-dimer and APTT are the traditional biomarkers that can be easily obtained to evaluate VTE in clinical practice.

Although there are few research studies and experimentation on the systemic procoagulant state caused by central nervous system tumors, these studies suggest that abnormal tumor microcirculation may release activated coagulation factors and procoagulant tissue factor containing microparticles (TF-MPs) into the periphery through a spillover effect, which leads to the activation of coagulation cascade [48]. We have found that a malignant CNS tumor (OR = 1.287, 95% CI [1.119, 1.480]) is an independent risk factor for postoperative VTE. The rise of procoagulant MPs is found in the blood of patients with recurrent glioblastoma [49]. However, a lower baseline level of tissue factor activity related to MPs in GBM cohorts or the expression of brain tumor-associated TF is indirectly associated with VTE [50,51]. Additional studies are needed to further explore the systemic procoagulant effects of these malignant CNS tumors. Considering that the diagnosis of malignant tumors should be based on postoperative pathology, the malignancy of the tumor cannot be accurately determined in the early postoperative period. Therefore, it was not included in the final model.

In our study, germinoma and acoustic neuroma were not identified as risk factors for postoperative VTE. Pituitary tumors, on the other hand, were found to be a protective pathological type for postoperative VTE in neurosurgery. This is likely due to the fact that most pituitary adenomas are managed through transnasal endoscopic surgery, which is associated with shorter surgical durations and rapid postoperative mobilization. Regarding the location of neural tumors, such as cerebral hemisphere, lateral ventricle, third ventricle, and cavernous sinus, as well as tumors located at the skull base, our study did not identify these as risk factors for postoperative VTE.

In our analysis and clinical practice, craniopharyngioma (CP) is a special tumor type with a higher incidence of VTE after craniopharyngioma resection than other common neurosurgical tumors. CP is a tumor that can likely be diagnosed preoperatively through cranial CT and MRI examinations in both axial and coronal planes. Senior neurosurgeons from our medical center have found that CP patients had a high incidence of VTE, and even fatal PE. A variety of comorbidities aggravates the occurrence of VTE after craniopharyngioma surgery, as well as prolonged postoperative immobilization and central venous catheters (CVCs) (OR = 1.346, 95% CI [1.166, 1.554] in univariable logistic regression of our study). Additionally, resection of recurrent craniopharyngiomas is difficult, which also prolongs surgery duration and increases the likelihood of damage to the surrounding vital anatomical structures, especially hypothalamus and pituitary stalk, leading to postoperative disorder of internal environment caused by central diabetes insipidus and electrolyte disturbances. The manifestations of hypothalamic syndrome and central diabetes insipidus are tractable weight gain or obesity, eating disorders, multiple neuroendocrine and pituitary deficiencies, and severe dehydration [52]. These are the risk factors for VTE [53,54,55]. For severe dehydration, inadequate desmopressin replacement therapy is also a risk factor for thrombosis [56]. Additionally, desmopressin administration induced the release of Von Willebrand factor multimers, which aggravated thrombophilia [57].

The neurosurgical operation may involve the vital structure of brain consciousness conduction. It may result in structural causes of postoperative disturbance of consciousness, such as bilateral cortical or diencephalic infarctions, edema, hematoma, acute lateral shift of the brain, and increased intracranial pressure that reduces cerebral blood flow (CBF) and infection. If the patient remains with disturbing consciousness on the second day after surgery, this leads to an increased incidence of postoperative VTE due to immobilization of their extremities and paralysis following intracerebral hemorrhage (ICH) [58]. In patients with disturbance of consciousness experiencing immobility and slurred speech, there should be increased vigilance for the occurrence of VTE.

The clinical utility of our model was evaluated using a decision-curve analysis. We found that it could be useful to predict those patients with a VTE risk greater than 0%–60% who would benefit from thromboprophylaxis. Neurosurgeons are concerned about postoperative intracranial or nonintracranial bleeding following the use of anticoagulants. Our nomogram might be used safely with the clinical prediction scales associated with bleeding.

Patients who undergo neurosurgical procedures are at high risk for VTE. The relevant guideline of the American College of Chest Physicians (ACCP) and the European Society of Anaesthesiology recommend that patients with a low risk of bleeding or a high risk of VTE should use regular postoperative pharmacologic prophylaxis alone or combine pharmacologic and mechanical methods of prophylaxis and delay initiation until at least 24 h after surgery. Patients with high bleeding risk are thought to be given only mechanical methods [13,59]. However, the American Society of Hematology (ASH) guideline panel suggests against using pharmacological prophylaxis ASH guidelines for patients undergoing major neurosurgical procedures [60]. Then, based on the support of the above guideline and this nomogram, within 72 h postneurosurgery, patients are stratified based on a cutoff value into high-risk (P_VTE_ > cutoff value) and low-risk (P_VTE_ < cutoff value) groups. For high-risk patients, they undergo basic prophylaxis + either pharmacological or combined pharmacological and mechanical prophylaxis. However, in cases of high-risk VTE patients with a substantial postoperative bleeding risk, we recommend basic prevention combined with mechanical prevention until the bleeding risk is significantly reduced. Low-risk patients receive basic prophylaxis combined with mechanical prophylaxis. Basic prophylaxis should recommend adopting bundles of care (Text S1 in Supplementary Materials S1). Mechanical prophylaxis includes graduated compression stockings (GCS), intermittent pneumatic compression devices (IPC), and venous foot pumps (VFPs). Currently, pharmacological prophylaxis after neurosurgery involves randomized controlled trial (RCT) intervention groups primarily using low molecular weight heparin or novel oral medications [61,62]. Pharmacological prophylaxis recommends using low molecular weight heparin (LMWH) or the novel oral anticoagulant rivaroxaban; LMWH: subcutaneous injection, once daily. The specific dosage and prophylactic contraindications are based on the drug’s instructions. For patients with severe renal impairment, heparin prophylaxis is recommended. Patients with a creatinine clearance < 30 mL/min should receive reduced doses. Rivaroxaban is recommended for single-drug prophylaxis, administered at least 24 h postoperation with a dosage of 10 mg orally once daily. It is suggested for prophylaxis for 7–14 days or until discharge.

Our primary outcome was a composite of DVT and PE after the neurosurgical procedure, so we cannot predict these outcomes separately. We did not include other coagulation parameters such as antithrombin levels in preoperative coagulation screening or postoperative coagulation tests. In the future, we intend to actively engage with the laboratory department. Additionally, we aim to include more coagulation parameters in further validation to enhance the depth of our research. Due to the limitations imposed by the neurosurgical indications included in our study, our clinical prediction model is currently applicable only to the indications described for neurosurgical patients listed in Table S2 in Supplementary Materials S1. We cannot guarantee its applicability for patients with other neurosurgical indications. Despite conducting prospective and external validation, we still hope that more medical centers will cooperate to verify this nomogram again.

## 5. Conclusions

In conclusion, a clinical prediction model for VTE after the neurosurgical procedure in hospitalization was developed and validated. Five clinical factors (age, craniopharyngioma, duration of operation, disturbance of consciousness on the second day after surgery, high dose of mannitol) and three biomarkers (D-dimer before surgery, APTT before neurosurgery, highest D-dimer within 72 h after surgery) were finally included to construct a nomogram and form an online calculator, and the model showed great discrimination, calibration, and clinical utility. Clinicians can further predict and evaluate the high-risk population of VTE in neurosurgery and give corresponding medical measures of the more standardized primary invention. Future endeavors aim to conduct further exploration on the risk factors delineated in this article and to refine clinical management based on these identified risk factors.

## Figures and Tables

**Table 1:** Development cohort (n = 4401), retrospective internal validation cohort (n = 1466), prospective internal validation cohort (n = 490), and external validation cohort (n = 2301) were assessed for baseline characteristics, candidate variables pre-, intra-, and post-operation, and outcome events *.

Baseline Characteristics and Variables	Development Cohort (n = 4401) †	Retrospective Internal Validation Cohort (n = 1466) †	Prospective Internal Validation Cohort (n = 490)	External Validation Cohort (n = 2301)
VTE events	1167 (26.52%)	390 (26.60%)	40 (8.16%)	104 (4.52%)
Preoperative				
Age (years), mean (SD)	50.42 (13.85)	50.08 (14.06)	48.64 (15.02)	49.08 (13.33)
Sex				
Male	2156 (49%)	753 (51.4%)	270 (55.7%)	1064 (46.2%)
Female	2245 (51%)	713 (48.6%)	215 (44.3%)	1237 (53.8%)
BMI (kg/m^2^), mean (SD)	24.72 (3.91)	24.62 (3.84)	25.04 (4.46)	24.59 (3.76)
KPS (score), mean (SD)	84.96 (18.9)	84.6 (19.95)	NA	NA
ASA				
1 or 2 level	4293 (97.5%)	1431 (97.6%)	NA	NA
3 or 4 or 5 level	465 (11.3%)	182 (13.3%)	NA	NA
Preoperative hospital stays (days), mean (SD)	5.85 (3.84)	5.78 (3.66)	NA	NA
Wheelchair or bedridden	108 (2.5%)	35 (2.4%)	NA	NA
Medical history				
Hypertension	841 (19.1%)	275 (18.8%)	NA	NA
Diabetes	388 (8.8%)	118 (8%)	NA	NA
Hyperlipidemia	170 (3.9%)	60 (4.1%)	NA	NA
Nephropathy	6 (0.1%)	3 (0.2%)	NA	NA
Hepatopathy	33 (0.7%)	16 (1.1%)	NA	NA
Varicosity	9 (0.2%)	4 (0.3%)	NA	NA
Preoperative diagnosis				
Intracranial aneurysm	158 (3.6%)	54 (3.7%)	NA	NA
Carotid artery stenosis	66 (1.5%)	26 (1.8%)	NA	NA
Trauma	35 (0.8%)	10 (0.7%)	NA	NA
Hydrocephalus	84 (1.9%)	24 (1.6%)	NA	NA
Spinal vascular malformation	11 (0.2%)	4 (0.3%)	NA	NA
Epilepsy	27 (0.6%)	8 (0.5%)	NA	NA
Trigeminal neuralgia	48 (1.1%)	14 (1%)	NA	NA
Hemifacial spasm	7 (0.2%)	1 (0.1%)	NA	NA
Brain abscess	39 (0.9%)	15 (1%)	NA	NA
Laboratory test results				
D-dimer (µg/mL), mean (SD)	0.66 (1.33)	0.84 (2.87)	0.94 (1.62)	2.89 (3.78)
Prothrombin time (s), mean (SD)	11.46 (1.11)	11.51 (0.92)	NA	NA
APTT (s), mean (SD)	25.57 (3.22)	25.41 (3.17)	27.81 (3.88)	23.9 (3.53)
Thrombin time (s), mean (SD)	17.75 (1.47)	17.74 (1.51)	NA	NA
Fibrinogen (g/L), mean (SD)	2.8 (0.87)	2.82 (0.97)	NA	NA
Prothrombin activity (%), mean (SD)	109.65 (22.52)	108.64 (21.44)	NA	NA
Hemoglobin (g/L), mean (SD)	133.15 (17.12)	133.2 (17.11)	NA	NA
Platelets (10^9^/L), mean (SD)	227.11 (65.63)	224.17 (62.82)	NA	NA
White blood cells (10^9^/L), mean (SD)	7.83 (4.8)	7.91 (4.73)	NA	NA
LDL (mmol/L), mean (SD)	3.06 (5.45)	2.94 (0.89)	NA	NA
Triglycerides (mmol/L), mean (SD)	1.62 (1.13)	1.6 (1.1)	NA	NA
Total cholesterol (mmol/L), mean (SD)	4.67 (1.12)	4.67 (1.15)	NA	NA
Uric acid (μmol/L), mean (SD)	313.55 (97.9)	315.79 (101.72)	NA	NA
ALT (U/L), mean (SD)	24.41 (25.04)	24.07 (21.92)	NA	NA
Na (mmol/L), mean (SD)	139.38 (3.08)	139.34 (3.22)	NA	NA
K (mmol/L), mean (SD)	3.98 (0.35)	3.98 (0.36)	NA	NA
Cl (mmol/L), mean (SD)	104.56 (3.54)	104.48 (3.66)	NA	NA
Serum homocysteine (umol/L), mean (SD)	14.94 (8.37)	14.96 (8.42)	NA	NA
Intraoperative				
Duration of operation (min), mean (SD)	263.73 (144.03)	265.41 (141.15)	230.22 (120.83)	202.88 (110.26)
Bleeding volume (mL), mean (SD)	444.49 (573.07)	449.6 (567.06)	NA	NA
Operation position (prone position)	258 (6.4%)	86 (6.5%)	NA	NA
Operation level				
3 level	519 (11.8%)	141 (9.6%)	NA	NA
4 level	3662 (83.5%)	1243 (84.9%)	NA	NA
Anesthesia method (general anesthesia)	4101 (99.2%)	1362 (99.3%)	NA	NA
Operative site				
Cerebellar hemisphere	119 (2.7%)	38 (2.6%)	NA	NA
Lateral ventricle	21 (0.5%)	7 (0.5%)	NA	NA
Fourth ventricle	27 (0.6%)	5 (0.3%)	NA	NA
Third ventricle	15 (0.3%)	2 (0.1%)	NA	NA
Cavernous sinus	109 (2.5%)	46 (3.1%)	NA	NA
Cranial base	109 (2.5%)	46 (3.1%)	NA	NA
Intraspinal	183 (4.2%)	51 (3.5%)	NA	NA
Intramedullary	116 (2.6%)	36 (2.5%)	NA	NA
Postoperative				
Highest D-dimer within 72 h ‖, mean (SD)	3.58 (4.47)	3.95 (5.35)	2.63 (3.86)	3.9 (5.83)
Disturbance of consciousness ‡	241 (8.8%)	92 (10%)	39 (8%)	21 (0.9%)
High dose of mannitol §	1230 (27.9%)	424 (28.9%)	151 (31.1%)	117 (5.1%)
CVC	1294 (29.4%)	399 (27.2%)	NA	NA
Lumbar cisterna drainage	481 (10.9%)	144 (9.8%)	NA	NA
Hemiplegia or Paraplegia	49 (1.1%)	21 (1.4%)	NA	NA
Malignant tumor ¶	1421 (34.7%)	483 (35.6%)	88 (18.1%)	NA
Secondary tumor ¶	259 (5.9%)	97 (6.6%)	19 (3.9%)	NA
Pituitary tumor ¶	423 (9.6%)	135 (9.2%)	NA	NA
Germinoma ¶	21 (0.5%)	14 (1%)	NA	NA
Acoustic neuromas ¶	257 (5.8%)	100 (6.8%)	NA	NA
Craniopharyngioma ¶	251 (5.7%)	71 (4.8%)	16 (3.3%)	216 (9.4%)

Abbreviations: VTE, venous thromboembolism. BMI, body mass index. KPS, Karnofsky performance status (KPS) scale. ASA, American Society of Anesthesiology score. AVM, arteriovenous malformations. APTT, activated partial thromboplastin time. LDL, low density lipoprotein. ALT, alanine aminotransferase. CVC, central venous catheterization. NA, not applicable. * Data represent n (%) or mean (standard deviation). † Randomization proportion for the development and retrospective internal validation sets was 3:1. ‡ Disturbance of consciousness on the second day after surgery. § High dose of mannitol represents the amount of 20% mannitol administered ≥ 250 mL intravenous drip q.8h. (daily dosage greater than 0.15 kg/24 h) for three days after surgery. ¶ Histology and malignancy of the tumor were evaluated according to the postoperative pathological reports. ‖ Highest D-dimer within 72 h after surgery is the highest value selected from the data tested immediately after surgery, the first day after surgery, the second day after surgery, and the third day after surgery.

**Table 2:** In the development cohort, a total of 67 candidate variables were analyzed for preoperative, intraoperative, and postoperative factors in neurosurgical VTE risk through their respective *p*-values in both univariate and multivariate logistic regression (MLR) analyses. The variables highlighted in bold are found to be significant in the MLR analysis.

Variables	Univariable LR		MLR	
	OR (95% CI)	*p*-Value *	OR (95% CI)	*p*-Value *
Preoperative variables				
**Age (years)**	1.047 (1.041–1.052)	<0.001	1.04 (1.033–1.047)	<0.001
Sex (Female)	1.104 (0.965–1.262)	0.149	NA	
BMI (kg/m^2^)	1.006 (0.989–1.024)	0.468	NA	
**KPS (score)**	0.985 (0.982–0.988)	<0.001	0.991 (0.987–0.996)	<0.001
**ASA (3 or 4 or 5 level)**	2.706 (2.238–3.271)	<0.001	1.411 (1.113–1.787)	0.004
Preoperative hospital stays (days)	1.041 (1.023–1.059)	<0.001	NA	
Wheelchair or bedridden	1.91 (1.283–2.842)	0.001	1.06 (0.649–1.712)	0.814
Medical history				
**Hypertension**	1.699 (1.446–1.996)	<0.001	1.221 (1.013–1.469)	0.036
Diabetes	1.349 (1.071–1.697)	0.011	NA	
Hyperlipidemia	1.152 (0.823–1.612)	0.411	NA	
Nephropathy	1.109 (0.215–5.722)	0.902	NA	
Hepatopathy	0.923 (0.45–1.894)	0.827	NA	
Varicosity	3.361 (1.024–11.034)	0.046	2.768 (0.619–11.567)	0.162
Preoperative diagnosis				
Intracranial AVM	1.414 (1.007–1.986)	0.046	0.83 (0.395–1.685)	0.614
Intracranial aneurysm	1.98 (1.442–2.718)	<0.001	1.879 (0.971–3.765)	0.067
Carotid artery stenosis	0.362 (0.164–0.799)	0.012	NA	
Trauma	1.517 (0.748–3.075)	0.248	NA	
Hydrocephalus	0.907 (0.545–1.51)	0.707	NA	
Spinal vascular malformation	1.188 (0.307–4.602)	0.803	NA	
Epilepsy	0.628 (0.237–1.663)	0.349	NA	
Trigeminal neuralgia	1.345 (0.724–2.501)	0.348	NA	
Hemifacial spasm	0.00 (0.00–1.57 × 10^133^)	0.943	NA	
Brain abscess	2.03 (1.062–3.878)	0.032	NA	
Laboratory test results				
**D-dimer (µg/mL)**	1.428 (1.332–1.531)	<0.001	1.12 (1.044–1.209)	0.003
Prothrombin time (s)	1.007 (0.948–1.07)	0.826	NA	
**APTT (s)**	0.919 (0.898–0.94)	<0.001	0.945 (0.921–0.969)	<0.001
Thrombin time (s)	0.982 (0.937–1.029)	0.439	NA	
Fibrinogen (g/L)	1.2 (1.116–1.29)	<0.001	0.989 (0.904–1.08)	0.801
Prothrombin activity (%)	1.001 (0.998–1.004)	0.415	NA	
Hemoglobin (g/L)	0.996 (0.992–1)	0.053	NA	
Platelets (10^9^/L)	1 (0.999–1.001)	0.596	NA	
White blood cells (10^9^/L)	1.025 (1.011–1.04)	0.001	1.012 (0.995–1.029)	0.165
LDL (mmol/L)	1.008 (0.995–1.022)	0.207	NA	
Triglycerides (mmol/L)	0.941 (0.88–1.007)	0.078	NA	
Total cholesterol (mmol/L)	1.114 (1.048–1.185)	0.001	0.992 (0.923–1.065)	0.822
Uric acid (μmol/L)	0.998 (0.997–0.998)	<0.001	NA	
ALT (U/L)	1.002 (0.999–1.005)	0.211	NA	
Na (mmol/L)	0.983 (0.962–1.005)	0.13	NA	
K (mmol/L)	0.784 (0.645–0.952)	0.014	NA	
Cl (mmol/L)	0.981 (0.962–1)	0.048	NA	
Serum homocysteine (umol/L)	1.003 (0.995–1.011)	0.483	NA	
Intraoperative variables				
**Duration of operation (min)**	1.003 (1.002–1.003)	<0.001	1.002 (1.002–1.003)	<0.001
Bleeding volume (mL)	1 (1–1)	<0.001	1 (1–1)	0.343
Operation position (prone position)	0.682 (0.498–0.935)	0.017	NA	
The operation level				
3 level	0.83 (0.67–1.03)	0.035	NA	
4 level	1.295 (1.069–1.569)	0.008	1.122 (0.882–1.435)	0.353
Anesthesia method (general anesthesia)	3.089 (1.094–8.724)	0.033	NA	
Operative site				
Cerebellar hemisphere	0.541 (0.337–0.868)	0.011	NA	
Lateral ventricle	1.214 (0.498–2.958)	0.67	NA	
Fourth ventricle	0.691 (0.259–1.847)	0.462	NA	
Third ventricle	0.923 (0.297–2.869)	0.891	NA	
Cavernous sinus	1.057 (0.7–1.598)	0.791	NA	
Cranial base	1.057 (0.7–1.598)	0.791	NA	
Intraspinal	0.891 (0.622–1.275)	0.527	NA	
Intramedullary	1.204 (0.791–1.833)	0.387	NA	
Postoperative variables				
**Highest_D_dimer_within_72_hours ‖**	1.208 (1.184–1.233)	<0.001	1.124 (1.101–1.148)	<0.001
**Disturbance of consciousness †**	3.363 (2.617–4.321)	<0.001	1.619 (1.195–2.192)	0.002
**High dose of mannitol ‡**	1.447 (1.253–1.67)	<0.001	1.79 (1.39–2.30)	<0.001
CVC	1.346 (1.166–1.554)	<0.001	0.969 (0.817–1.146)	0.712
Lumbar cisterna drainage	1.472 (1.2–1.806)	<0.001	NA	
Hemiplegia or Paraplegia	1.351 (0.754–2.421)	0.312	NA	
Malignant tumor §	1.287 (1.119–1.48)	<0.001	NA	
Secondary tumor §	1.042 (0.792–1.371)	0.769	NA	
Pituitary tumor §	0.467 (0.353–0.617)	<0.001	NA	
Germinoma §	0.24 (0.056–1.017)	0.053	NA	
Acoustic neuromas §	0.709 (0.522–0.965)	0.029	NA	
**Craniopharyngioma §**	1.948 (1.499–2.531)	<0.001	2.348 (1.709–3.219)	<0.001

Abbreviations: LR, logistic regression. MLR, multivariable logistic regression. BMI, body mass index. KPS, Karnofsky performance status (KPS) scale. ASA, American Society of Anesthesiology score. AVM, arteriovenous malformations. APTT, activated partial thromboplastin time. LDL, low density lipoprotein. ALT, alanine aminotransferase. CVC, central venous catheterization. NA, not applicable. * *p*-value < 0.05 was considered statistical significance. † Disturbance of consciousness on the second day after surgery. ‡ High dose of mannitol represents the amount of 20% mannitol administered ≥ 250 mL intravenous drip q.8h. for three days after surgery. § Histology and malignancy of the tumor were evaluated according to the postoperative pathological reports. ‖ Highest D-dimer within 72 h is the highest value selected from the data tested immediately after surgery, the first day after surgery, the second day after surgery, and the third day after surgery.

## Data Availability

The datasets used and/or analyzed during the current study are available from the corresponding author on reasonable request.

## Supplementary Materials

The following supporting information can be downloaded at: https://www.mdpi.com/article/10.3390/cancers15225483/s1. Supplementary Materials S1, Figure S1: Flowcharts (a) Flowcharts for the development cohort and retrospective internal validation cohort. (b) Flowchart for the prospective internal validation cohort. (c) Flowchart for the external validation cohort. Figure S2: Graphical representation of the variables’ contribution to the three axis of the PCA. Table S1: Details of the reagents used. Table S2: A comprehensive description of the surgical indications for all patients in each neurosurgery cohort. Table S3: In the entire retrospective cohort, univariable logistic regression was utilized to calculate the odds ratios (ORs) representing the correlation between various types of neurosurgical procedures and the occurrence of postoperative VTE. Text S1: Content of Bundled Care. Supplementary Materials S2, TRIPOD Checklist: Prediction Model Development and Validation.
