# Supplementary material for: Development and Validation of a Clinical Prediction Model for Venous Thromboembolism Following Neurosurgery: A 6-Year, Multicenter, Retrospective and Prospective Diagnostic Cohort Study
Source: Cancers (Basel). 2023 Nov 20;15(22):5483. doi: 10.3390/cancers15225483 (PMC10670076; doi:10.3390/cancers15225483)
Supplement: Supplementary file 1 [file cancers-15-05483-s001.zip › Supplementary Material S1.pdf]

Supplementary Material S1 Content

Figure S1 Flowcharts (a) Flowcharts for the development cohort and retrospective internal validation cohort. (b) Flowchart for the prospective internal validation cohort. (c) Flowchart for the external validation cohort.

Figure S2 Graphical representation of the variables' contribution to the three axis of the PCA

Table S1 Details of the reagents used

Table S2 A comprehensive description of the surgical indications for all patients in each neurosurgery cohort

Table S3 In the entire retrospective cohort, univariable logistic regression was utilized to calculate the odds ratios (ORs) representing the correlation between various types of neurosurgical procedures and the occurrence of postoperative VTE

Text S1 Content of Bundled Care

Figure S1 Flowcharts (a) Flowcharts for the development cohort and retrospective internal validation cohort. (b) Flowchart for the prospective internal validation cohort. (c) Flowchart for the external validation cohort.

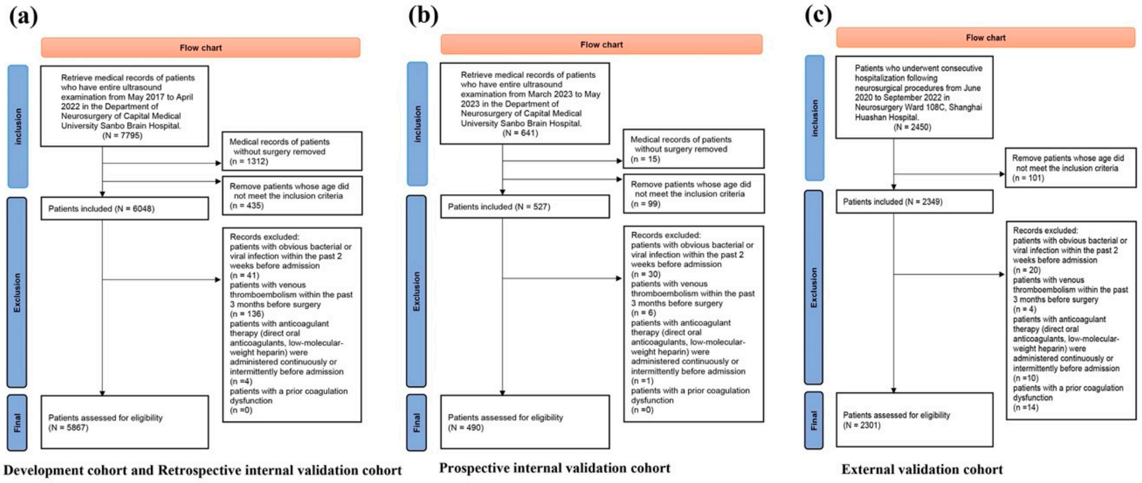

Figure S2 Graphical representation of the variables' contribution to the three axis of the PCA

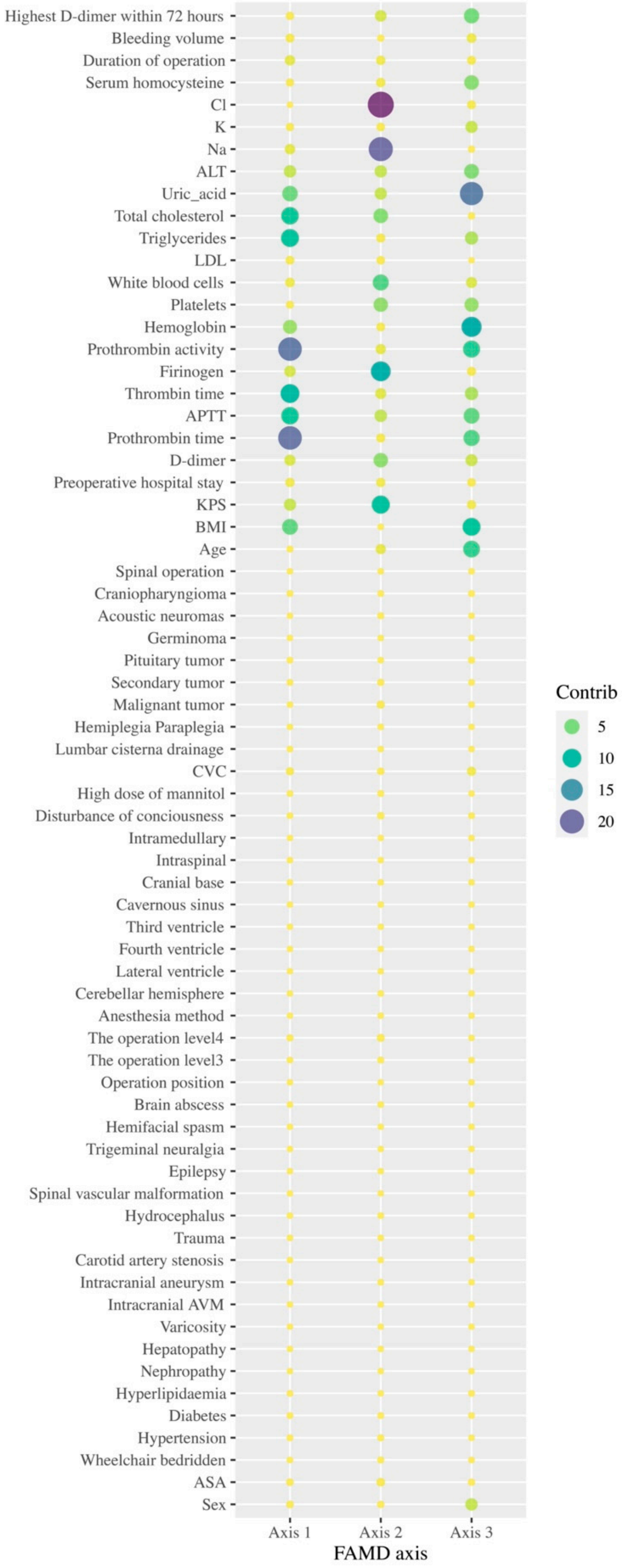

Table S1 Details of the reagents used

Laboratory test items	English names of reagent	Reagent detection method	Company	Company's country
D-dimer (ug/mL)[a]	INNOVANCE D-Dimer	Immunoassay method	Sysmex	Japan
Prothrombin time (s)	Thromborel S	Clotting method	Sysmex	Japan
APTT (s)[a]	Dade Actin FSL Activated PTT	Clotting method	Sysmex	Japan
Thrombin time (s)	Test Thrombin	Clotting method	Sysmex	Japan
Fibrinogen (g/L)	Dade Thrombin	Clotting method	Sysmex	Japan
Prothrombin activity (%)	Thromborel activity	Clotting method	Sysmex	Japan
Hemoglobin (g/L)	hemoglobin	Colorimetry method	Mindray	China
Platelets (109/L)	platelet	Impedance method	Mindray	China
White blood cells (109/L)	leukocyte	Impedance method	Mindray	China
LDL (mmol/L)	LDL-Cholesterol	End-point method	Beckman	America
Triglycerides (mmol/L)	Triglyceride	End-point method	Beckman	America
Total cholesterol (mmol/L)	Cholesterol	End-point method	Beckman	America
Uric acid (μmol/L)	Uric Acid	Uricase method	Leadman	China
ALT (U/L)	Alanine Aminotransferase	Rate method	Beckman	America
Na (mmol/L)	Sodium	ISE method-indirect	Beckman	America
K (mmol/L)	Potassium	ISE method-indirect	Beckman	America
Cl (mmol/L)	Chlorine	ISE method-indirect	Beckman	America
Serum homocysteine (umol/L)	Homocysteine	Cyclic enzymatic method	Leadman	China

[a]Shanghai Huashan Hospital used the same reagents as Beijing Sanbo brain Hospital.

Table S2 A comprehensive description of the surgical indications for all patients in each neurosurgery cohort

The indications for all neurosurgical patients in each cohort	Development cohort (n=4401)	Retrospective internal validation cohort (n=1466)	Prospective internal validation cohort (n=490)	External validation cohort (n=2301)
Neuro-oncology (brain)	2258 (51.3%)	891 (60.8%)	289 (59.0%)	2271 (98.7%)
Cerebrovascular neurosurgery for aneurysms, arteriovenous malformations (AVMs), stroke, and other related conditions	1203 (20.5%)	262 (17.9%)	83 (16.9%)	NA
Functional and epilepsy neurosurgery, trigeminal neuralgia and nerve compression syndromes	490 (11.1%)	107 (7.3%)	28 (5.7%)	NA
Spinal neurosurgery	591 (13.4%)	146 (10.0%)	39 (8.0%)	NA
Other neurosurgery	160 (3.64%)	60 (4.1%)	51 (10.4%)	30 (1.3%)

Table S3 In the entire retrospective cohort, univariable logistic regression was utilized to calculate the odds ratios (ORs) representing the correlation between various types of neurosurgical procedures and the occurrence of postoperative VTE.

The indications for all neurosurgical patients in the entire retrospective cohort	Univariable LR	
	OR (95% CI)	P value*
Neuro-oncology (brain)	1.037 (0.898-1.197)	0.622
Cerebrovascular neurosurgery for aneurysms, arteriovenous malformations (AVMs), stroke, and other related conditions	1.596 (1.261-2.019)	<0.001
Functional and epilepsy neurosurgery, trigeminal neuralgia and nerve compression syndromes	1.011 (0.727-1.405)	0.950
Spinal neurosurgery	0.722 (0.567-0.919)	0.008

*P value<0.05 was considered statistical significance.

Text S1 Content of Bundled Care

Basic prophylaxis should recommend adopting bundled care, including: (1) establishment of a bundled care team; (2) assessment of DVT risk in patients; (3) psychological guidance; (4) health education primarily pre-operation, with continual reinforcement post-operation. Patients are guided on in-bed hygiene practices, emphasizing the importance of early ambulation in VTE prophylaxis, encouraged to exercise post-operation, such as elevating legs, maintaining fluid balance, avoiding dehydration. For non-ambulatory patients, their family members are advised to perform gastrocnemius and soleus muscle compression from the Achilles tendon upwards; (5) pain intervention, advising patients to divert their attention, thereby increasing pain threshold and adhering to analgesics.
